# Administration of glycyrrhetinic acid reinforces therapeutic effects of mesenchymal stem cell‐derived exosome against acute liver ischemia‐reperfusion injury

**DOI:** 10.1111/jcmm.15675

**Published:** 2020-09-09

**Authors:** Xiaolin Wei, Wenjing Zheng, Peikai Tian, Hui Liu, Yong He, Minjie Peng, Xiangde Liu, Xiaowu Li

**Affiliations:** ^1^ Department of Hepatobiliary Surgery Shenzhen University General Hospital Shenzhen University Clinical Medical Academy Shenzhen China; ^2^ Department of Hepatobiliary Surgery Southwest Hospital Third Military Medical University (Army Medical University) Chongqing China

**Keywords:** Glycyrrhetinic acid, HMGB1, Inflammation, IR, MSCs, TLR4

## Abstract

Recent studies have shown that mesenchymal stem cell‐derived exosome could attenuate ischaemia‐reperfusion (I/R) injury by suppressing inflammatory response in the liver. Glycyrrhetinic acid was also shown to be capable of repressing the TLR4 signalling pathway. However, it remains to be explored as whether the combined administration of mesenchyma stem cell (MSC)‐derived exosome and glycyrrhetinic acid (GA) could increase their therapeutic effects on I/R injury. Western blot was performed to evaluate the expression of proteins associated with inflammatory response in THP‐1 cells and I/R rat models treated under different conditions. Flow cytometry was carried out to analyse the proportions of different subtypes of peripheral blood cells in I/R rats. Alanine aminotransferase (ALT) and aspartate aminotransferase (AST) were measured to assess the liver injury in I/R rats. Combined treatment with MSC‐derived exosome and GA effectively maintained the expression of key proteins involved in inflammatory response in LPS stimulated THP‐1 cells and THP‐1 cells treated under hypoxia conditions. In the established of I/R rat models, GA administration reinforced the therapeutic efficiency of MSC‐derived exosomes by maintaining the proportion of different subgroups of peripheral blood cells, decreasing the concentration of ALT and AST, and restoring the expression of dysregulated proteins associated with inflammation. Our results demonstrated that treatment with exosomes derived from mesenchymal stem cells (MSCs) attenuated liver I/R injury, while the pre‐treatment with GA may further promote the therapeutic effect of mesenchymal stem cell‐derived exosome against acute liver ischaemia‐reperfusion injury.

## 1 INTRODUCTION

The injury of liver ischaemia‐reperfusion (LIR) is seen under numerous circumstances consisting of hypovolemic shock from dehydration, extended cardiopulmonary cerebral resuscitation (CPCR), septic shock, as well as upon significant hepatic resection or when total severance of vascular supply to the liver is needed.^1^, ^2^ LIR injury has actually been recognized as a significant reason of hepatic failures. Although the critical care technique has seen major advancements, patients of LIR injury continue to suffer from severe hepatic failures and high mortality.^1^, ^2^, ^3^, ^4^ Consequently, it is important to both medical professionals as well as clinical scientists to discover a risk‐free and also effective method to treat patients with hepatic failures refractory to traditional therapy.

Mesenchymal stem cells (MSC) are cells which can be separated from numerous types of tissues, including the umbilical cord, bone marrow, adipose tissues, liver, peripheral blood, as well as tooth root.^5^, ^6^ Also, MSC transplantation provides trophic support to the I/R‐injured liver by inhibiting hepatocellular apoptosis and by stimulating regeneration.^7^ These cells can adhere to plastic culture plates so that they can be passaged for nearly 50 generations while still maintaining their multi‐potency.^8^, ^9^ Exosomes derived from MSC were initially studied in mouse myocardial I/R injury and then studied afterwards using in a number of disease models.^10^, ^11^ Previous research revealed that exosomes can play a cardio‐protective role in paracrine secretion, highlighting the impact of MSC‐derived exosomes on tissue and organ repair.^10^

Toll‐like receptors (TLRs) are present in lots of cell types, specifically in cells of innate immunity, to identify infection as well as harmful foreign intruders. Many TLR analogues have actually been found, such as TLR4, which was actually extensively explored in numerous illnesses.^12^, ^13^ TLR4 plays a role specific to cell types in liver I/R injury.^14^, ^15^ Specially, grafting bone marrow‐derived cells from TLR4 wild‐type mice into TLR4 mutant mice increased the damage to the retina, which indicated an essential role for TLR4 in bone marrow‐derived cells contributing to the progression of diabetic retinopathy.^15^ Nevertheless, the leading outcome of TLR4 in liver I/R injury is to cause damages by inducing inflammatory reaction to molecules including high mobility group box 1.^16^

As an aglycone saponin isolated from glycyrrhizae radix, glycyrrhetinic acid (GA) supposedly displays a variety of medicinal results, consisting of anti‐inflammatory, anti‐tumour as well as hepato‐protective effects.^17^, ^18^, ^19^, ^20^ It was actually shown that GA is hepato‐protective in numerous liver diseases, such as liver damages induced by CCl4, hepatic failures induced by LPS/D‐GalN, hepatotoxicity induced by triptolides, as well as liver damages induced by free fatty acids.^17^ Although GA was reported to play a protective role in I/R injury of heart by lowering the sensitivity as well as occurrence of ventricular arrhythmia, it was revealed that GA pre‐treatment can dramatically subdue the expression of p‐ERK, HMGB1, p‐p38, p‐JNK, as well as pIRAK1 in the liver.^21^ These outcomes showed that GA prevented liver injury induced by APAP and the reason may be related to prevent the secretion of HMGB1 as well as to activate the NFκB/TLR4‐IRAK1‐MAPK signalling.^22^ Previous research has actually verified that the suppression of neutrophils can relieve APAP‐induced liver injuries.^23^, ^24^ Due to the fact that IL‐1β and TNF‐α are downstream of TLR4 signalling, GA was actually shown to suppress TLR4 signalling. As anticipated, GA hindered the expression of IL‐1β and TNF‐α in the liver in the presence of APAP. Similarly, it was suggested that APAP dramatically set off substantial macrophage infiltration as well as neutrophil recruitment, but GA substantially reduced the effect of APAP, showing that GA undermined HMGB1 dependent inflammatory reactions in mice exposed to APAP.^22^

It has been shown that MSC‐derived exosomes may alleviate liver I/R injury by suppressing inflammatory response in the liver, while GA was shown to suppress the signalling pathway of TLR4.^25^, ^26^ In this study, we pre‐treated I/R cells and animals with MSC‐derived exosomes and GA to study their effects on the liver reperfusion injury and inflammatory response in the liver.

## 2 MATERIALS AND METHODS

### 2.1 Animal and treatment

A rat I/R (Ischaemia/Reperfusion) model was established with a total of 40 male SD rats weighting between 210 to 255 g. In brief, rats were purchased from our animal centre and divided into 4 groups, that is, 1. NC (N = 10, sham treated rats); 2. I/R (N = 10, I/R rats); 3. I/R + EXO (N = 10, I/R rats treated with exosomes alone); 4. I/R + EXO +GA (N = 10, I/R rats treated with exosomes plus 100 mg/kg of GA by intraperitoneal shot). For the establishment of I/R group, non‐lethal I/R was caused by anesthetizing the rats first with 100 mg/kg of Phenobarbital. Then, midline laparotomy was carried out by occluding the portal venous as well as arterial blood supply to the centre as well as left lobes of liver by using atraumatic clamps. After 90 minutes of reperfusion, the clamps were released for 6 hours of reperfusion before the cut was sutured. For the establishment of I/R + EXO +GA group, the rats were treated using 100 mg/kg of GA (pureness level of >99%, Nanjing Zelang, Nanjing, China) given via intraperitoneal injections prior to the surgical procedure. The rats in the NC group got an equivalent quantity of olive oil. All rats were killed after 6 hours of reperfusion, and their liver and serum samples were gathered for further evaluation. Institutional ethical committee has approved the protocol of this study.

### 2.2 In vitro study for assessment of anti‐inflammatory and anti‐oxidative properties of GA

The anti‐inflammatory and anti‐oxidative activities of GA were evaluated by making use of a specialized testing kit (Thermo Fisher Scientific, Waltham, MA). In brief, 150 μL of assay buffer as well as 10 μL of sample were added to every well. After 5 minutes of incubation at ambient temperature, 20 μL of reaction buffer was loaded into each well. After another 5 minutes of incubation at ambient temperature, the signal was read on a BioTek microplate reader (BioTek, Winooski, VT, USA) at the absorbance level of 590 nm.

### 2.3 ADMSC‐derived exosomes

The aim of making use of MSC‐derived exosomes for the therapy of rat LIR injury was to validate the inherent ability of xenogenic exosomes to prevent inflammation as well as oxidative stress. The specific objectives were as follows: (a) to identify the suppressive role of exosomes in inflammatory response, 1.0 × 10^5^ of RAW 264.7 macrophage cells (ATCC, Virginia, MA) were cultured for thirty minutes with 100 ng/mL of lipopolysaccharides. The cells were cleansed with PBS and then co‐cultured for 6 or 12 hours with 50 µmol/L of exosomes. (b) To evaluate the influence of exosomes as well as melatonin on apoptosis as well as oxidative stress, hepatocytes were treated for 3 hours with exosomes or melatonin and then cultured for 12 hours under hypoxic conditions in 5% carbon dioxide, 1% O2, as well as 94% N2 in a hypoxia chamber. The exosomes were isolated following routines provided in by a exosome extraction kit (Thermo Fisher Scientific, Waltham, MA, USA).

### 2.4 Cell culture and transfection

THP‐1 cells were purchased from ATCC (Virginia, MA, USA) and randomly divided into 4 groups, that is, 1. NC group (control cells); 2. LPS group (cells treated with 100 ng/mL of LPS for thirty minutes); 3. LPS + EXO group (cells treated with 100 ng/mL of LPS and MSC‐derived exosomes for 24 hours); and 4. LPS + EXO +GA group (cells treated with 100 ng/mL of LPS, MSC‐derived exosomes and 25 μmol/L GA for 24 hours). In another experiments, THP‐1 cells were randomly divided into 4 groups, 1. NC group (control cells); 2. HYPOXIA group (cells cultured in 5% carbon dioxide, 1% O2, as well as 94% N2); 3. HYPOXIA + EXO group (cells cultured in 5% carbon dioxide, 1% O2, as well as 94% N2 and treated with MSC‐derived exosomes for 24 hours); 4. HYPOXIA + EXO +GA group (cells cultured in 5% carbon dioxide, 1% O_2_, as well as 94% N2 and treated with MSC‐derived exosomes and 25 μmol/L of GA for 24 hours).

### 2.5 Flow cytometry

The percentages of Annexin V^+^/PI^−^, Annexin V^+^/ PI^+^, and CD3^+^/CD4^+^ cells in collected peripheral blood mononuclear cells (PBMNC) and splenocytes, as well as the percentage of CD3^+^/CD8^+^ cells in collected PBMNC/splenocytes, were measured by flow cytometry using commercial assay kits (Thermo Fisher Scientific) in accordance with the instructions provide by the manufacturer.

### 2.6 Detection of liver ALT and AST levels

Samples of venous blood were collected into blood collection vacuum tubes that contained an anticoagulant. Then, the venous blood samples were centrifuged at 1500 *g* within 1 hour after sample collection to separate the serum component. Then, the levels of ALT and AST in each sample were determined by making use of an AU 680 bioanalyzer (Beckman Coulter, San Jose, CA, USA).

### 2.7 Western blot analysis

Complete cell lysate was isolated by using a RIPA lysis buffer added with 45 mmol/L of β‐mercaptoethanol, 10 mmol/L of pH 8.0 Tris, 5 mmol/L of EGTA, 100 mmol/L of NaCl, 1% of NP 40, 0.1% of SDS, 50 mmol/L of NaF, 10 mg/mL of aprotinine, 1 mmol/L of PMSF as well as 10 mg/mL of leupeptin. Then, the lysate was incubated for 30 minutes on ice before being filtered through a membrane filter and centrifuged for 15 minutes at 14 000 *g* and 4°C. Then, protein samples (containing 30 μg of protein in each sample) were resolved by 10% SDS‐PAGE, blotted onto a nitrocellulose membrane, treated with primary antibodies against TLR4, HMGB1, IL‐1b, TNF‐a, c‐CASP‐3, c‐PARP, NOX1, NOX‐2, c‐CASP‐3, c‐PARP, and Bcl‐2, incubated with corresponding secondary antibodies, developed using an ECL reagent (Thermo Fisher Scientific) in accordance with the instructions provide by the manufacturer and evaluated to calculate the relative protein expression of TLR4, HMGB1, IL‐1b, TNF‐a, c‐CASP‐3, c‐PARP, NOX1, NOX‐2, c‐CASP‐3, c‐PARP, and Bcl‐2 in each sample. All antibodies were used at the dilution of 1:10 000 and were supplied by Abcam (Cambridge, UK).

### 2.8 Statistical analysis

The study results were analysed by utilizing SPSS 22 software (SPSS, IBM, Armonk, NY, USA). All results were shown as mean ± standard deviation (SD). To examine the differences between different groups, Student's t test was utilized for comparisons. A two‐tailed *P* value of <0.05 was deemed statistically significant.

## 3 RESULTS

### 3.1 Combined treatment with MSC‐derived exosomes and GA prevented LPS induced up‐regulation of TLR4, HMGB1, IL‐1b and TNF‐a expression in THP‐1 cells

**FIGURE 1:**
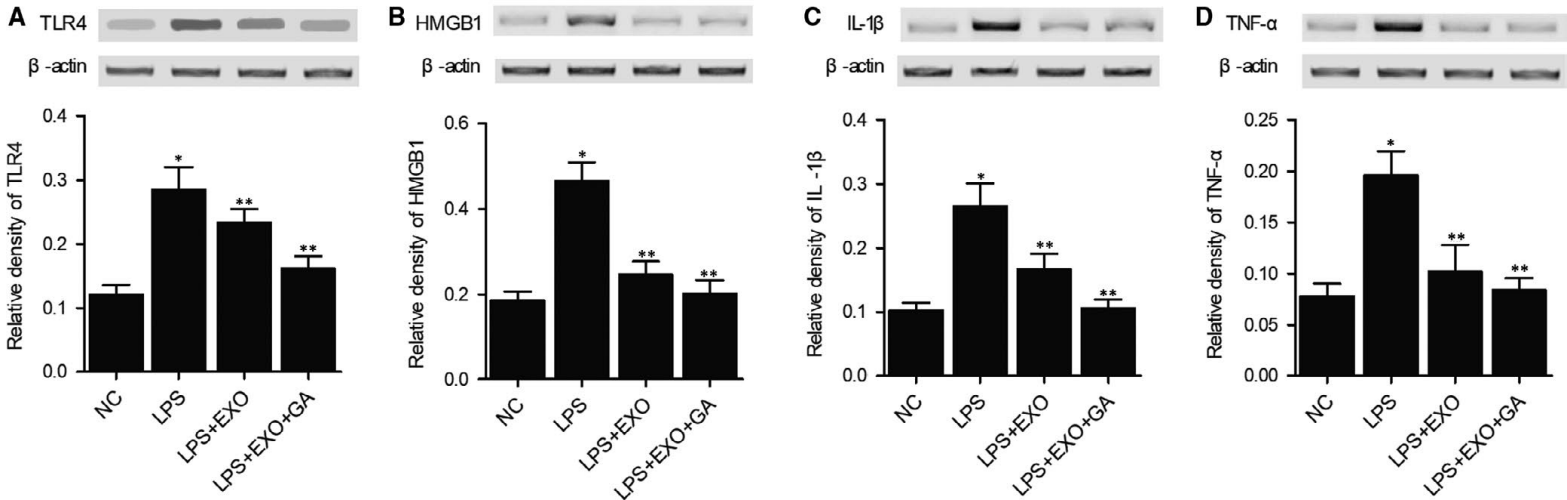
Aberrant up‐regulation of TLR4, HMGB1, IL‐1b and TNF‐a in LPS stimulated THP‐1 cells was restored to normal by MSC‐derived exosomes and GA (*P value < 0.05 vs NC group; **P value < 0.05 vs LPS group). A, Aberrant up‐regulation of TLR4 in LPS stimulated THP‐1 cells was restored to normal by MSC‐derived exosomes and GA. B, Aberrant up‐regulation of HMGB1 in LPS stimulated THP‐1 cells was restored to normal by MSC‐derived exosomes and GA. C, Aberrant up‐regulation of IL‐1b in LPS stimulated THP‐1 cells was restored to normal by MSC‐derived exosomes and GA. D, Aberrant up‐regulation of TNF‐a in LPS stimulated THP‐1 cells was restored to normal by MSC‐derived exosomes and GA

THP‐1 cells were treated with LPS and subjected to further treatment with MSC (Mesenchymal stem cell)‐derived exosomes alone or in combination with GA (Glycyrrhetinic acid). Western blot was performed to analyse the expression of TLR4 (Figure 1A), HMGB1 (Figure 1B), IL‐1b (Figure 1C) and TNF‐a (Figure 1D) proteins in the THP‐1 cells treat under different conditions. The expression of TLR4, HMGB1, IL‐1b and TNF‐a proteins was significantly increased in THP‐1 cells treated with LPS. Treatment with MSC‐derived exosomes alone showed considerable effects to suppress LPS stimulated up‐regulation of TLR4, HMGB1, IL‐1b and TNF‐a in THP‐1 cells. Moreover, combined treatment with MSC‐derived exosomes and GA almost restored the normal expression of TLR4, HMGB1, IL‐1b and TNF‐a proteins. These results indicated that GA treatment had a positive effect on enhancing the capability of MSC‐derived exosomes.

### 3.2 Combined treatment with MSC‐derived exosomes and GA prevented hypoxia induced up‐regulation of c‐CASP‐3, c‐PARP, NOX1 and NOX2 protein expression in THP‐1 cells

**FIGURE 2:**
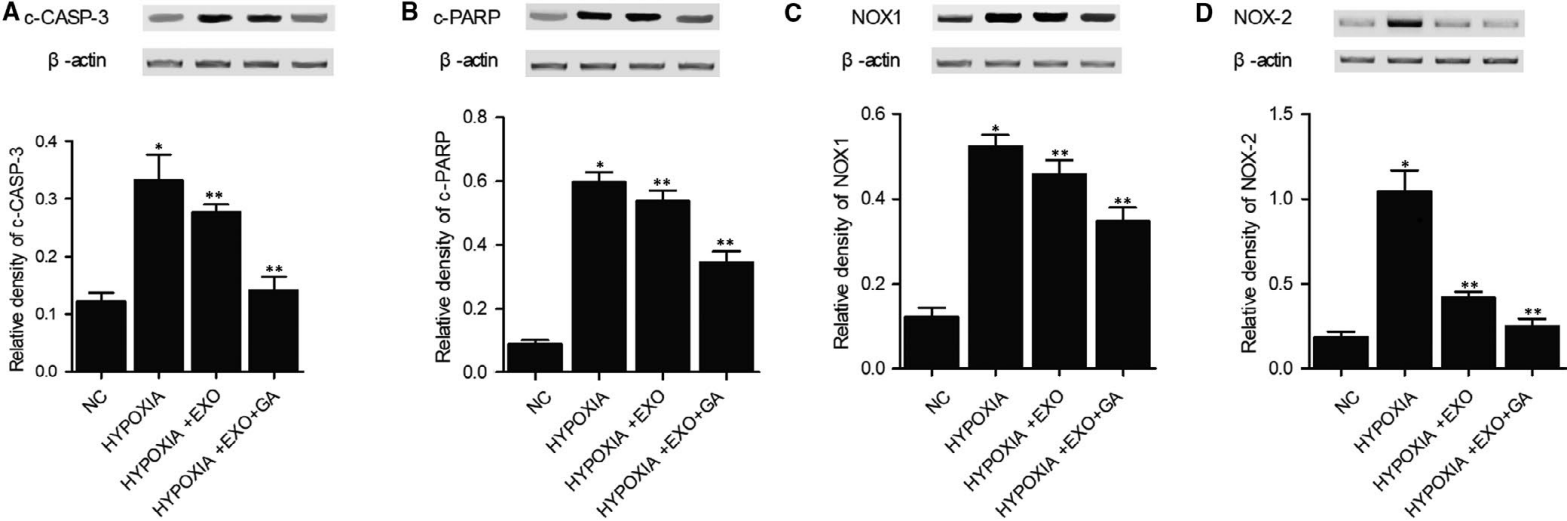
Abnormal increase of c‐CASP‐3, c‐PARP, NOX1 and NOX2 protein expression in THP‐1 cells treated under hypoxia conditions was restored to normal by MSC‐derived exosomes and GA (*P value < 0.05 vs NC group; **P value < 0.05 vs LPS group). A, Abnormal up‐regulation of c‐CASP‐3 in LPS stimulated THP‐1 cells was restored to normal by MSC‐derived exosomes and GA. B, Abnormal up‐regulation of c‐PARP in LPS stimulated THP‐1 cells was restored to normal by MSC‐derived exosomes and GA. C, Abnormal up‐regulation of NOX1 in LPS stimulated THP‐1 cells was restored to normal by MSC‐derived exosomes and GA. D, Abnormal up‐regulation of NOX2 in LPS stimulated THP‐1 cells was restored to normal by MSC‐derived exosomes and GA

THP‐1 cells were cultured under a hypoxia condition and subjected to further treatment with MSC‐derived exosomes alone or in combination with GA. Expression of c‐CASP‐3 (Figure 2A), c‐PARP (Figure 2B), NOX1 (Figure 2C) and NOX2 (Figure 2D) proteins was measured using Western blot. Hypoxia caused dramatic elevation of c‐CASP‐3, c‐PARP, NOX1 and NOX2 protein expression in THP‐1 cells, and MSC‐derived exosomes attenuated the expression of c‐CASP‐3, c‐PARP, NOX1 and NOX2 proteins to a certain extent. Combined treatment with MSC‐derived exosomes and GA further decreased the expression of c‐CASP‐3, c‐PARP, NOX1 and NOX2 proteins in THP‐1 cells treated under the hypoxia condition.

### 3.3 Combined treatment with MSC‐derived exosomes and GA maintained the proportions of Annexin V^+^/PI^−^, Annexin V^+^/PI^+^, CD3^+^/CD4^−^, CD3^+^/CD4^+^, CD3^+^/CD8^+^ cells in PBMNCs and CD3^+^/CD8^+^ cells in I/R rats

**FIGURE 3:**
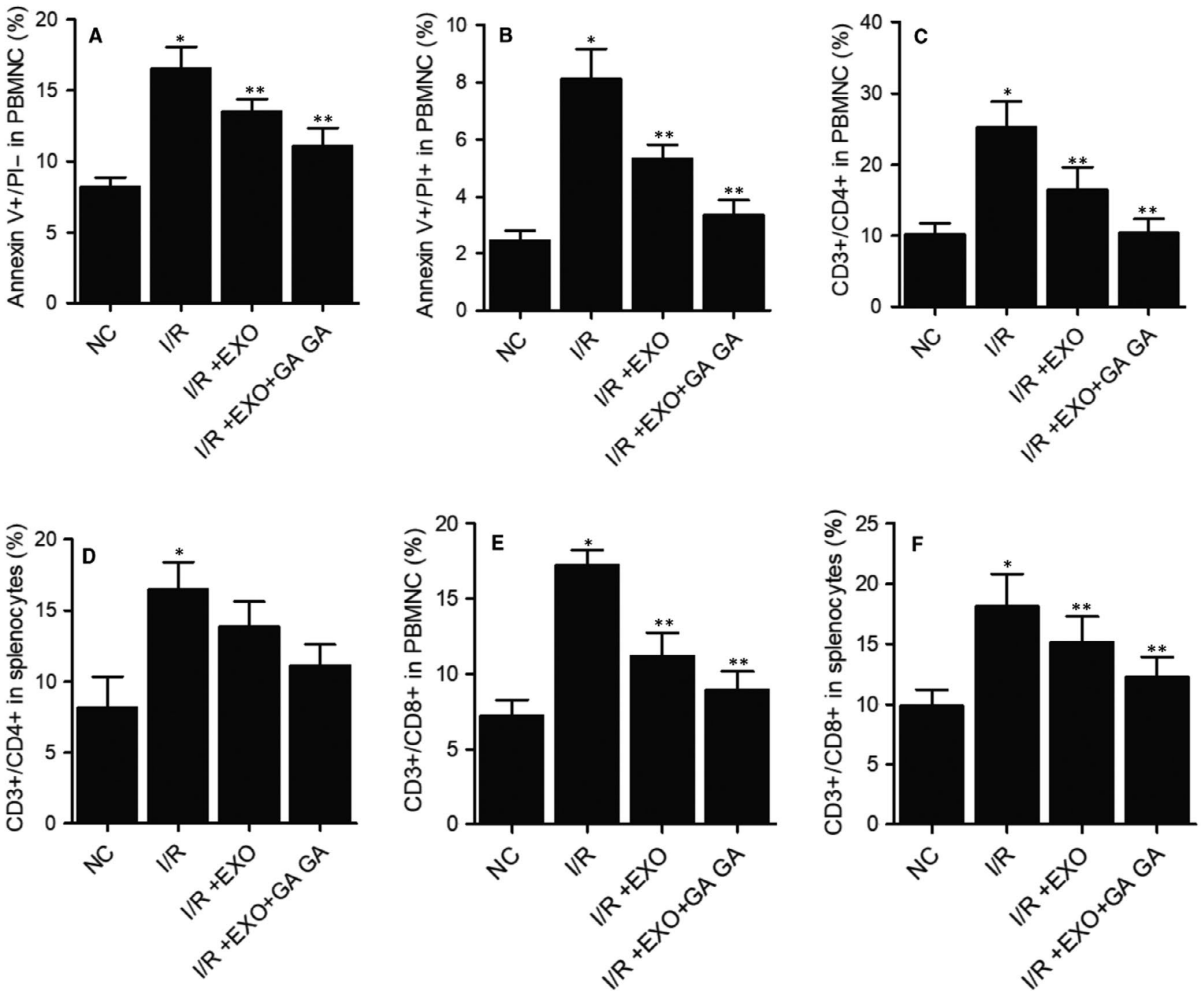
MSC‐derived exosomes and GA restored the normal proportions of different cell subpopulations in the peripheral blood of I/R rats (*P value < 0.05 vs NC group; **P value < 0.05 vs I/R group). A, The elevated percentage of Annexin V^+^/PI^−^ cells was restored to normal by combined treatment with MSC‐derived exosomes and GA. B, The elevated percentage of Annexin V^+^/PI^+^ cells in PBMNCs was restored to normal by combined treatment with MSC‐derived exosomes and GA. C, The elevated percentage of CD3^+^/CD4^−^ cells in PBMNCs was restored to normal by combined treatment with MSC‐derived exosomes and GA. D, The elevated percentage of CD3^+^/CD4^+^ cells in PBMNCs was restored to normal by combined treatment with MSC‐derived exosomes and GA. E, The elevated percentage of CD3^+^/CD8^+^ cells in PBMNCs was restored to normal by combined treatment with MSC‐derived exosomes and GA. F, The elevated percentage of CD3^+^/CD8^+^ cells in splenocytes was restored to normal by combined treatment with MSC‐derived exosomes and GA

A rat I/R (Ischaemia/Reperfusion) model was established as described and subjected to treatment with MSC‐derived exosomes alone or in combination with GA. Flow cytometry was performed to evaluate the percentages of Annexin V^+^/PI^−^, Annexin V^+^/PI^+^, CD3^+^/CD4^−^, CD3^+^/CD4^+^, and CD3^+^/CD8^+^ cells in PBMNCs and CD3^+^/CD8^+^ cells in splenocytes of I/R rats treated under different conditions. As shown in Figure 3, the percentages of Annexin V^+^/PI^−^ (Figure 3A), Annexin V^+^/PI^+^ (Figure 3B), CD3^+^/CD4^−^ (Figure 3C), CD3^+^/CD4^+^ (Figure 3D), and CD3^+^/CD8^+^ (Figure 3E) cells in PBMNCs and CD3^+^/CD8^+^ (Figure 3F) cells in splenocytes were remarkably elevated in I/R rats. Combined treatment with MSC‐derived exosomes and GA showed the highest efficiency to maintain the normal proportions of Annexin V^+^/PI^−^, Annexin V^+^/PI^+^, CD3^+^/CD4^−^, CD3^+^/CD4^+^, and CD3^+^/CD8^+^ cells in PBMNCs and CD3^+^/CD8^+^ cells in splenocytes.

### 3.4 Combined treatment with MSC‐derived exosomes and GA maintained the normal levels of ALT and AST in the plasma of I/R rats

**FIGURE 4:**
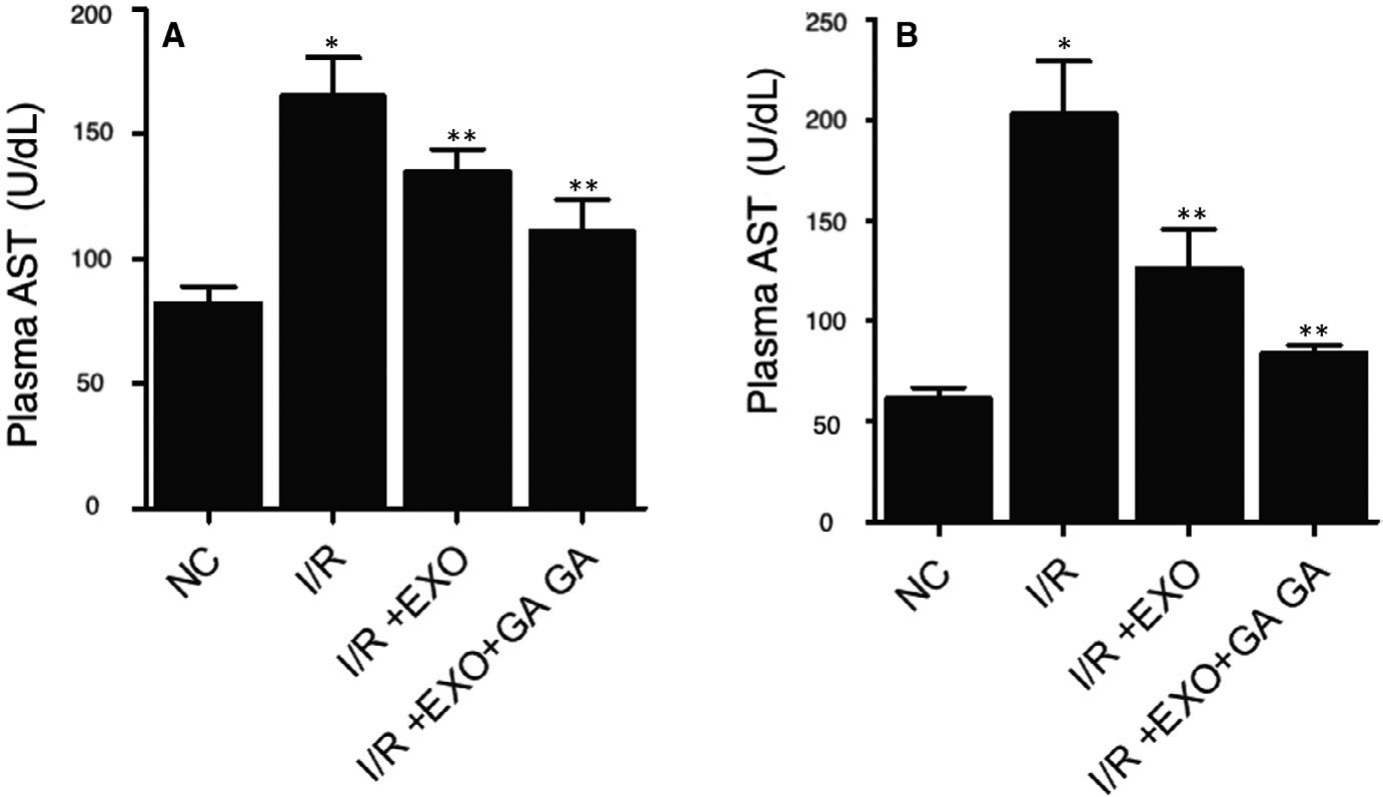
The alleviated ALT and AST concentrations in I/R rats were suppressed by combined treatment with MSC‐derived exosomes and GA (*P value < 0.05 vs NC group; **P value < 0.05 vs I/R group). A, The alleviated ALT concentration in I/R rats was suppressed by combined treatment with MSC‐derived exosomes and GA. B, The alleviated AST concentration in I/R rats was suppressed by combined treatment with MSC‐derived exosomes and GA

Furthermore, the levels of ALT (Figure 4A) and AST (Figure 4B) were examined in I/R rats. The concentrations of ALT and AST in the plasma of I/R rats were notably elevated. Combined treatment with MSC‐derived exosomes and GA showed high efficiency to repress the levels of ALT and AST in the plasma of I/R rats. These results demonstrated that the treatment with MSC‐derived exosomes and GA could reverse the liver injury caused by I/R.

### 3.5 Combined treatment with MSC‐derived exosomes and GA maintained the normal expression of NOX1, NOX2, c‐CASP‐3, c‐PARP, Bcl‐2, TLR4, IL‐1b, TNF‐a, NF‐kB, HMGB1 and IL‐10 proteins in I/R rats

**FIGURE 5:**
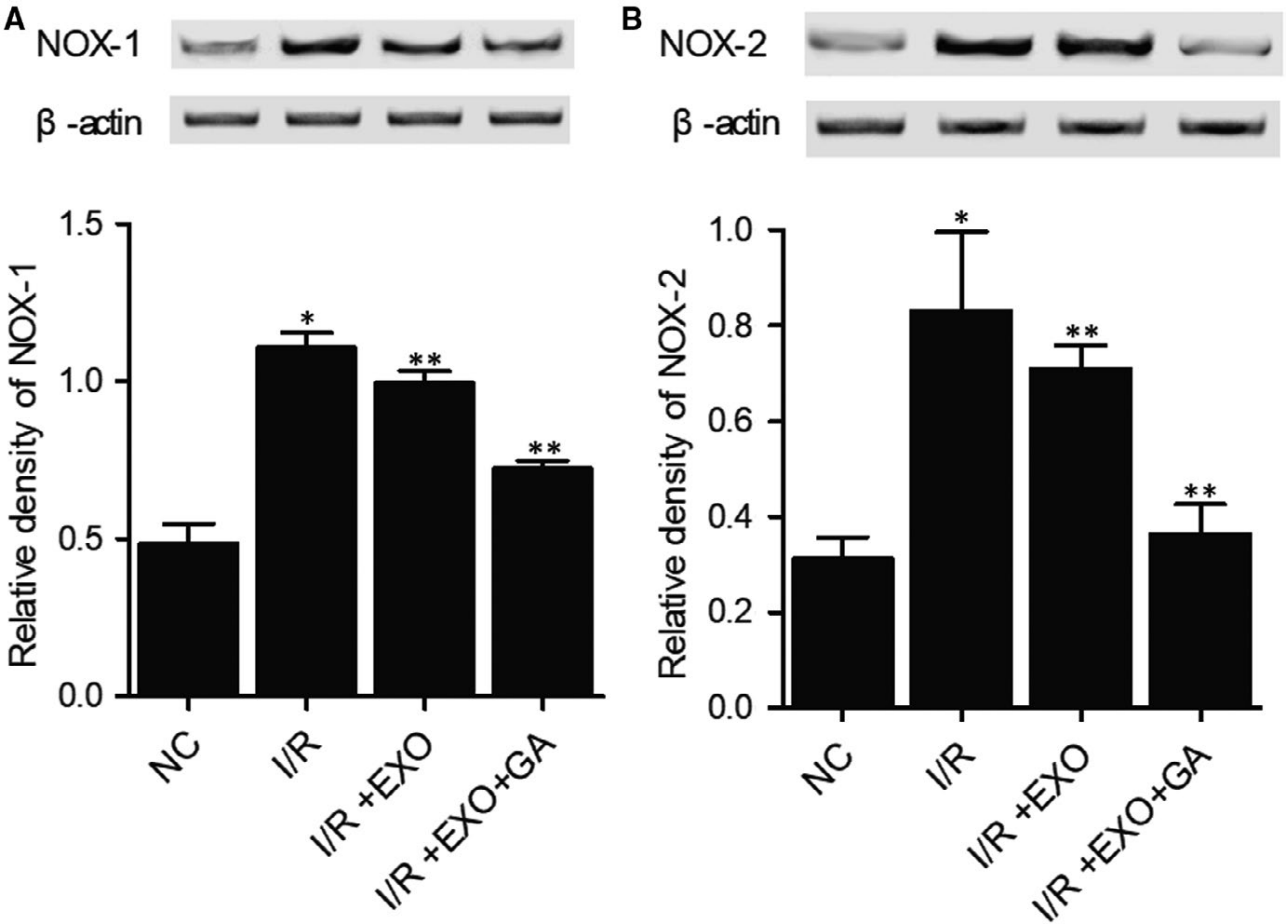
Abnormal increase of NOX1 and NOX2 protein expression in I/R rats was restored to normal by MSC‐derived exosomes and GA (*P value < 0.05 vs NC group; **P value < 0.05 vs I/R group). A, Abnormal up‐regulation of NOX1 in I/R rats was restored to normal by MSC‐derived exosomes and GA. B, Abnormal up‐regulation of NOX2 in I/R rats was restored to normal by MSC‐derived exosomes and GA

**FIGURE 6:**
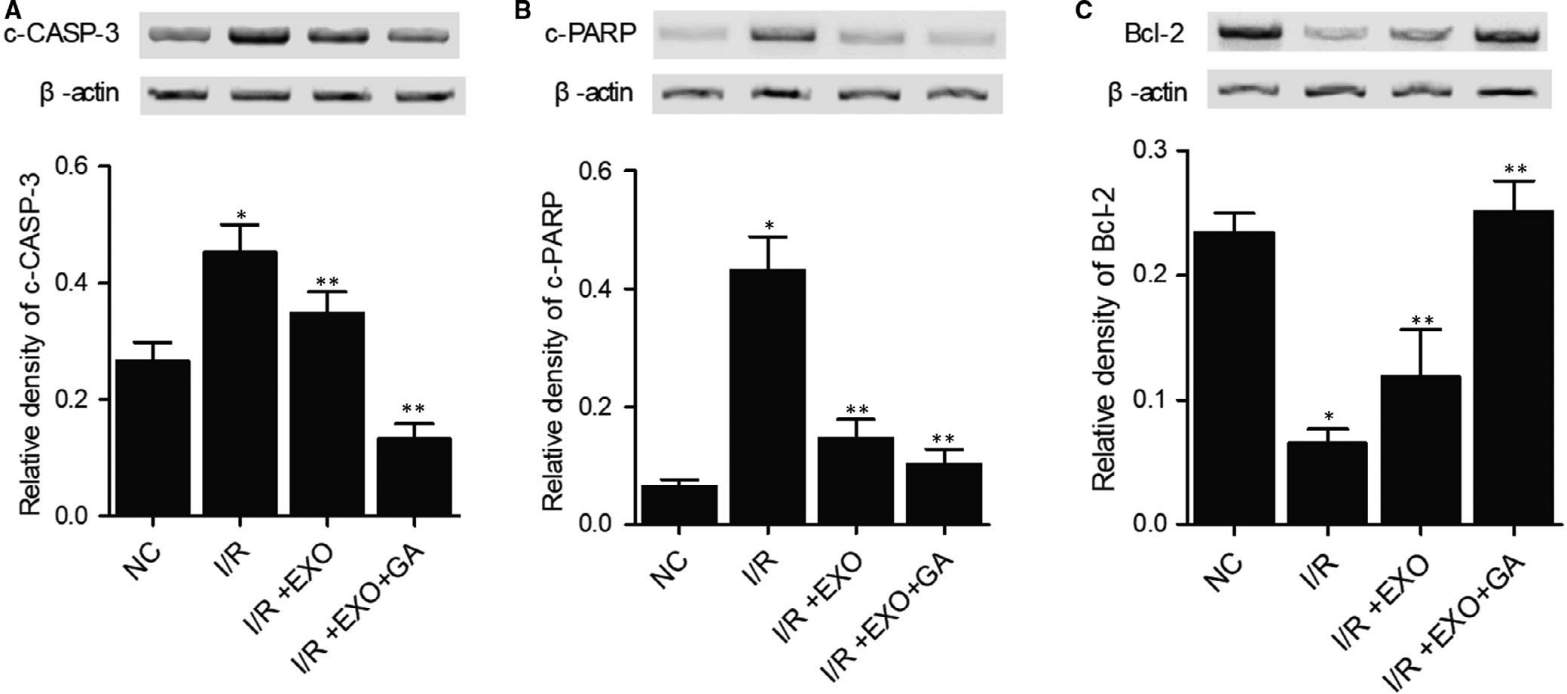
Dysregulation of c‐CASP‐3, c‐PARP and Bcl‐2 in I/R rats was restored to normal by combined treatment with MSC‐derived exosomes and GA (*P value < 0.05 vs NC group; **P value < 0.05 vs I/R group). A, Aberrant up‐regulation of c‐CASP‐3 in I/R rats was restored to normal by combined treatment with MSC‐derived exosomes and GA. B, Aberrant up‐regulation of c‐PARP in I/R rats was restored to normal by combined treatment with MSC‐derived exosomes and GA. C, Aberrant down‐regulation of Bcl‐2 in I/R rats was restored to normal by combined treatment with MSC‐derived exosomes and GA

**FIGURE 7:**
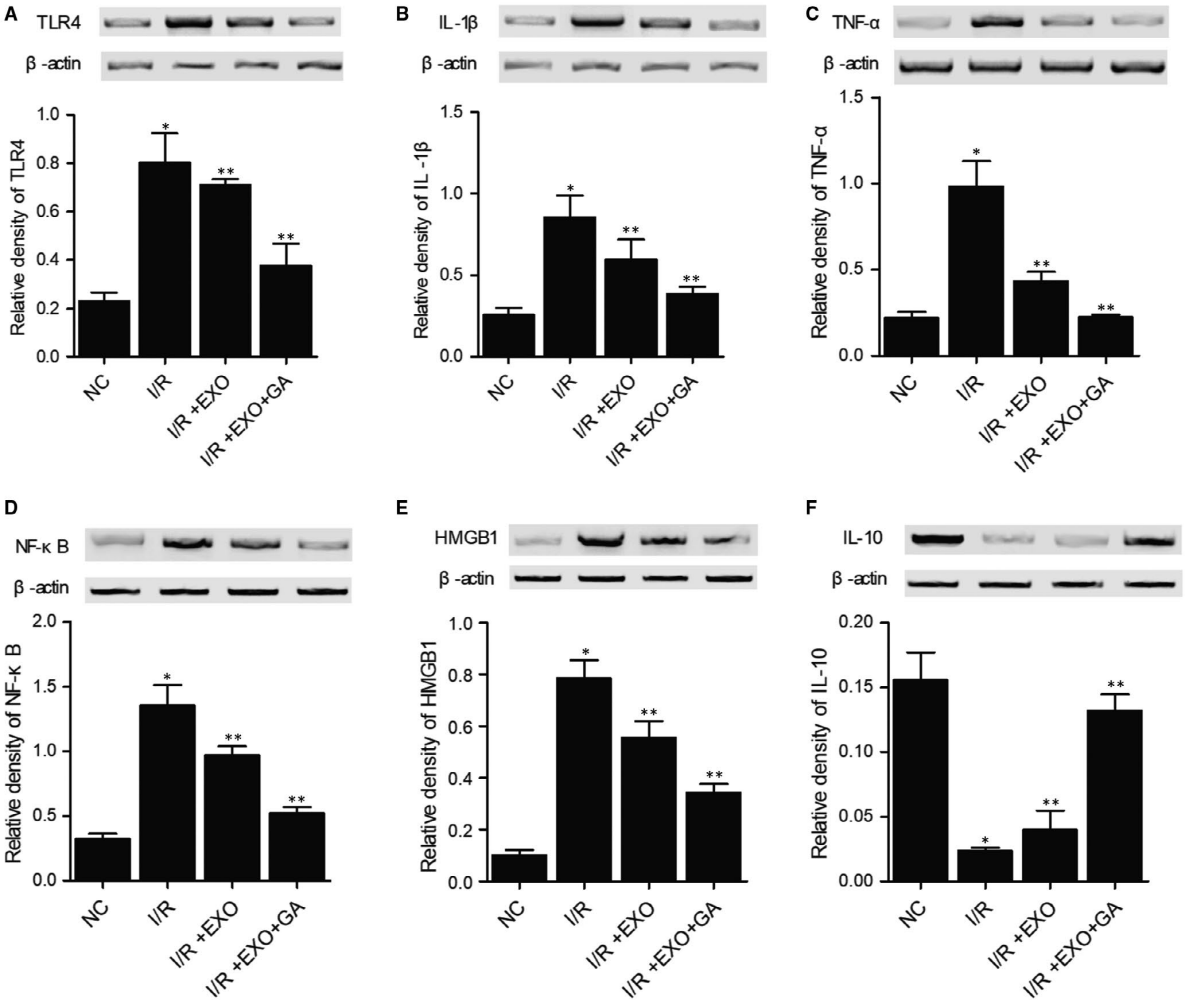
Dysregulation of TLR4, IL‐1b, TNF‐a, NF‐kB, HMGB1 and IL‐10 in I/R rats was restored to normal by combined treatment with MSC‐derived exosomes and GA (*P value < 0.05 vs NC group; **P value < 0.05 vs I/R group). A, Abnormal elevation of TLR4 expression in I/R rats was restored to normal by combined treatment with MSC‐derived exosomes and GA. B, Abnormal elevation of IL‐1b expression in I/R rats was restored to normal by combined treatment with MSC‐derived exosomes and GA. C, Abnormal elevation of TNF‐a expression in I/R rats was restored to normal by combined treatment with MSC‐derived exosomes and GA. D, Abnormal elevation of NF‐kB expression in I/R rats was restored to normal by combined treatment with MSC‐derived exosomes and GA. E, Abnormal elevation of HMGB1 expression in I/R rats was restored to normal by combined treatment with MSC‐derived exosomes and GA. F, Abnormal repression of IL‐10 expression in I/R rats was restored to normal by combined treatment with MSC‐derived exosomes and GA

We further analysed the expression of some key proteins involved in inflammatory response to evaluate the therapeutic effects of MSC‐derived exosomes and GA on I/R rats. The expression of NOX1 (Figure 5A), NOX2 (Figure 5B), c‐CASP‐3 (Figure 6A), c‐PARP (Figure 6B), TLR4 (Figure 7A), IL‐1b (Figure 7B), TNF‐a (Figure 7C), NF‐kB (Figure 7D) and HMGB1 (Figure 7E) proteins was evidently increased in I/R rats. On the contrary, the expression of Bcl‐2 (Figure 6C) and IL‐10 (Figure 7F) proteins was remarkably decreased in I/R rats. Treatment with MSC‐derived exosomes obviously reversed the abnormal up‐regulation of NOX1, NOX2, c‐CASP‐3, c‐PARP, TLR4, IL‐1b, TNF‐a, NF‐kB, and HMGB1 proteins and down‐regulated Bcl‐2 and IL‐10 proteins in I/R rats. Moreover, the combined treatment with MSC‐derived exosomes and GA almost fully restored the normal expression of these key proteins in I/R rats. These results demonstrated that GA treatment could enhance the capability of MSC‐derived exosomes in attenuating the inflammatory response in I/R rats.

## 4 DISCUSSION

Mesenchymal stem cells (MSC) are derived from bone marrow in adults and have actually become one of the most encouraging types of stem cells for dealing with heart disease.^27^ Although the restorative impact of MSCs has actually been credited to their ability to differentiate to reparative cells such as endothelial cells, cardiomyocytes, as well as vascular smooth cells, it was actually shown that a few types of such cells are moderated by paracrine factors produced by MSC.^28^, ^29^, ^30^, ^31^ The exosomes originated from MSC prevent liver I/R by reducing inflammatory reactions, reducing the amount of apoptotic factors like caspase‐3 and boosting the amount of anti‐oxidative factors including glutathione peroxidase (GSH‐Px) as well as glutathione (GSH).^32^ MSC‐derived exosomes might communicate with several kinds of nearby as well as remote cells to evoke certain feedbacks.^33^ Compared to other exosomes, MSC‐derived exosomes can carry nucleic acids, lipids as well as proteins. Using mass spectrometry as well as microarray evaluation, near 5000 distinct gene products and more than 4000 miRNAs were found in MSC‐derived exosomes.^34^ These exosomal miRNAs and proteins are functionally complicated and also are linked to numerous cellular as well as biochemical activities, including cell‐cell interaction, bioenergetics, immune regulation, tissue repair as well as metabolic process. In this study, we established a rat I/R model to evaluate the therapeutic effects of MSC‐derived exosomes and GA. MSC‐derived exosomes and GA restored the normal proportions of Annexin V^+^/PI^−^, Annexin V^+^/PI^+^, CD3^+^/CD4^−^, CD3^+^/CD4^+^, and CD3^+^/CD8^+^ cells in PBMNCs and CD3^+^/CD8^+^ cells in splenocytes, and reversed the liver injury caused by I/R. In addition, we carried out Western blot to evaluate the expression of proteins related to inflammatory response in I/R rats treated under different conditions. Combined treatment with MSC‐derived exosomes and GA effectively restored the normal expression of proteins related to inflammatory response in I/R rats.

Glycyrrhiza radix was long utilized as a natural medication for the therapy of liver disease, peptic ulcer, as well as skin diseases.^35^ Among the bioactive substances present in Glycyrrhiza radix, glycyrrhetinic acid (GA) is produced from glycyrrhizic acid (GL) via the hydrolyzation induced by glucuronidases.^36^ Medicinal research revealed that these substances had the ability to prevent the duplication of a number of RNA as well as DNA viruses involved in systemic and hepatic infections.^37^ It was discovered that GA had the ability to promote TLR‐4 expression while activating its downstream pathway. Located on cell surface, TLR‐4 can bind to viral ligands, microbial LPS, as well as protozoan particles to play essential roles in virus recognition and the synthesis of chemokines and cytokines.^38^ In this study, we treated LPS stimulated THP‐1 cells with MSC‐derived exosomes and GA, and then performed Western blot to evaluate the expression of TLR4, HMGB1, IL‐1b and TNF‐a. LPS stimulation up‐regulated TLR4, HMGB1, IL‐1b and TNF‐a proteins, which were then down‐regulated by the combined treatment with MSC‐derived exosomes and GA. Furthermore, we cultured THP‐1 cells under a hypoxia condition and treated them with MSC‐derived exosomes and GA. The expression of c‐CASP‐3, c‐PARP, NOX1 and NOX2 proteins was reduced by the combined treatment with MSC‐derived exosomes and GA.

Glycyrrhetinic acid was studied for its interaction with HMGB1.^39^ Lately, GA was shown to exhibit substantial toxicity in tumours of the central nerve system, suggesting that the targeting of HMGB1 with GA may be used for regulating the growth as well as development of cancers.^40^, ^41^, ^42^

TLR4 might be involved in taxol resistance by means of binding to MD2.^43^, ^44^ Lipopolysaccharides, the natural TLR4 ligand, are associated with innate immunity during microbial infection.^45^ TLR4 results in IRF3, MAPK as well as NFκB activation to increase the expression of pro‐inflammatory cytokines and INF‐γ through MyD88 pathways.^46^, ^47^ Liver IR entails complicated communication consisted of the activation of Kupffer cells, the production of reactive oxygen species, as well as neutrophil infiltration, eventually leading to the apoptosis of endothelial cells as well as hepatocytes yy.^48^, ^49^, ^50^ However, there are limitations of this study. Although the findings were validated in cellular and animal models, the further application and clinic value should be investigated, preferably in patients with acute liver ischaemia‐reperfusion injury.

## 5 CONCLUSION

In conclusion, the findings of this study demonstrated that the treatment with MSC‐derived exosomes alleviated liver I/R injury, and the pre‐treatment with GA may further promote the therapeutic effects of mesenchymal stem cell‐derived exosomes on the treatment of acute liver ischaemia‐reperfusion injury.

## CONFLICT OF INTEREST

None.

## AUTHOR CONTRIBUTION

**Xiaolin Wei:** Data curation (lead); Formal analysis (equal). **Wenjing Zheng:** Methodology (lead); Resources (equal); Software (equal). **Peikai Tian:** Conceptualization (equal); Data curation (equal); Resources (equal). **Hui Liu:** Data curation (equal); Formal analysis (equal); Investigation (equal); Methodology (equal). **Yong He:** Conceptualization (equal); Resources (equal); Software (equal); Visualization (equal). **Minjie Peng:** Formal analysis (equal); Investigation (equal); Validation (equal). **Xiangde Liu:** Data curation (equal); Funding acquisition (equal); Resources (equal); Software (equal); Supervision (equal); Visualization (equal); Writing‐original draft (equal). **Xiaowu Li:** Data curation (equal); Formal analysis (equal); Funding acquisition (equal); Resources (equal); Supervision (equal); Writing‐original draft (equal).

## Data Availability

The data that support the findings of this study are available from the corresponding author upon reasonable request.
